# Development of an Inactivated Vaccine against SARS CoV-2

**DOI:** 10.3390/vaccines9111266

**Published:** 2021-11-02

**Authors:** Shaikh Terkis Islam Pavel, Hazel Yetiskin, Muhammet Ali Uygut, Ahmet Furkan Aslan, Günsu Aydın, Öznur İnan, Büşra Kaplan, Aykut Ozdarendeli

**Affiliations:** 1 Department of Microbiology, Medical Faculty, Erciyes University, Kayseri 38280, Turkey; biotech.pavel@outlook.com (S.T.I.P.); hazelyetiskin@gmail.com (H.Y.); afaslan95@gmail.com (A.F.A.); gunsuaydinn@gmail.com (G.A.); busra.kaplan.3@gmail.com (B.K.); 2 Vaccine Research, Development and Application Center, Erciyes University, Kayseri 38280, Turkey; mauygut@gmail.com; 3 İstanbul Experimental Research Development and Education Center (İDEA), Istanbul 34303, Turkey; oznurinan.vet@gmail.com

**Keywords:** SARS-CoV-2, vaccine, inactivated vaccine, immunogenicity, ERUCoV-VAC, COVID-19 vaccine, TURKOVAC

## Abstract

The rapid spread of SARS-CoV-2 with its mutating strains has posed a global threat to safety during this COVID-19 pandemic. Thus far, there are 123 candidate vaccines in human clinical trials and more than 190 candidates in preclinical development worldwide as per the WHO on 1 October 2021. The various types of vaccines that are currently approved for emergency use include viral vectors (e.g., adenovirus, University of Oxford/AstraZeneca, Gamaleya Sputnik V, and Johnson & Johnson), mRNA (Moderna and Pfizer-BioNTech), and whole inactivated (Sinovac Biotech and Sinopharm) vaccines. Amidst the emerging cases and shortages of vaccines for global distribution, it is vital to develop a vaccine candidate that recapitulates the severe and fatal progression of COVID-19 and further helps to cope with the current outbreak. Hence, we present the preclinical immunogenicity, protective efficacy, and safety evaluation of a whole-virion inactivated SARS-CoV-2 vaccine candidate (ERUCoV-VAC) formulated in aluminium hydroxide, in three animal models, BALB/c mice, transgenic mice (K18-hACE2), and ferrets. The hCoV-19/Turkey/ERAGEM-001/2020 strain was used for the safety evaluation of ERUCoV-VAC. It was found that ERUCoV-VAC was highly immunogenic and elicited a strong immune response in BALB/c mice. The protective efficacy of the vaccine in K18-hACE2 showed that ERUCoV-VAC induced complete protection of the mice from a lethal SARS-CoV-2 challenge. Similar viral clearance rates with the safety evaluation of the vaccine in upper respiratory tracts were also positively appreciable in the ferret models. ERUCoV-VAC has been authorized by the Turkish Medicines and Medical Devices Agency and has now entered phase 3 clinical development (NCT04942405). The name of ERUCoV-VAC has been changed to TURKOVAC in the phase 3 clinical trial.

## 1. Introduction

COVID-19 is caused by a novel positive-strand RNA coronavirus (SARS-CoV-2) belonging to the Coronaviridae family, along with severe acute respiratory syndrome (SARS) and the Middle East respiratory syndrome (MERS) coronavirus [1]. The transcription of coronaviruses requires a polymerase template switch, characterised by a discontinuous process unique among RNA [2,3,4]. The SARS-CoV-2 RNA genome is approximately 30 kb and encodes four structural proteins: nucleocapsid (N), membrane (M), envelope (E), and spike (S) proteins, and its genome encodes 16 non-structural (nsp1–nsp16) and several accessory proteins [5,6]. The SARS-CoV-2 virus initiates the infection of the S protein into the human body through its interaction with the human angiotensin-converting enzyme 2 (ACE2) [7].

At the end of 2019, coronavirus disease 2019 (COVID-19) was first identified in Wuhan, a city in the Hubei province of China [8]. Due to the rapid spread of SARS-CoV-2 and the lack of pre-existing immunity, COVID-19 has posed a great threat to public health and safety [9].

The World Health Organization (WHO) declared the outbreak of COVID-19 to be a Public Health Emergency of International Concern on 30 January 2020 and officially recognized it as a pandemic on 11 March 2020. As of 4 October 2021, SARS-CoV-2 has infected more than 234 million people and has caused more than 4.8 million deaths (WHO dashboard, https://covid19.who.int/, accessed 4 October 2021).

Multiple control measures have been taken by the global community to cope with the current outbreak; these include wearing a medical mask; maintaining social distancing; performing hand hygiene; and COVID-19-specific management, including remdesivir/therapeutic antibodies and dexamethasone (WHO/2019-nCoV/clinical/2020.5) [10,11,12]. However, confirmed cases of COVID-19 have continued to rise around the world. This has become a primary health threat for humankind and has severely impacted the economic as well as the social situation [13]. Globally, scientists have focussed on developing various types of vaccines to combat this outbreak, which include live attenuated vaccines, protein subunit vaccines, viruses-like particles (VLP), viral vector-based vaccines, DNA vaccines, mRNA vaccines, and whole inactivated vaccine candidates [14,15,16,17,18]. Among these, whole inactivated vaccines are the most commonly used types for human and veterinary vaccines [19]. However, variation exists in the role of these vaccines in combatting different viral proteins. The rapid spread of SARS-CoV-2 with its mutating strains during the COVID-19 pandemic reinforces the crucial need for generating efficient and safe vaccines to prevent and control the spread of the virus.

A suitable animal model for COVID-19 is critical for the preclinical evaluation of the safety and efficacy of vaccine candidates. Animal models that could adequately simulate the viral infection and its development similarly to that in humans are regarded as a perfect choice to investigate the performance of vaccines. Some of the animal models that were used earlier to assess the productiveness of vaccines in preclinical trials include mice, rats, hamsters, guinea pigs, and ferrets [19,20,21,22]. Laboratory strains of mice are not susceptible to infection with SARS-CoV-2 since mouse angiotensin-converting enzyme 2 (mACE2) is incapable of efficiently binding to the SARS-CoV-2. Several non-human primate (NHP) animal models have also been developed for the study of COVID-19 with varying susceptibility of the host to SARS-CoV-2 infection [23,24]. However, the severity level of clinical manifestations in these models only ranges from mild to moderate, which makes it difficult to assess the efficacy of vaccines. It is important that the animal models that recapitulate the severe and fatal progression evaluate the efficacy of COVID-19 vaccine candidates. Transgenic mice (K18-hACE2) expressing the human SARS-CoV-2 receptor human angiotensin-converting enzyme (hACE2) under a cytokeratin 18 (K-18) promoter are susceptible to SARS-CoV-2, and infection resulted in a lethal disease course [25,26]. Therefore, the K18-hACE2 mouse model has been useful for vaccine challenge studies.

We previously reported the isolation of the hCoV-19/Turkey/ERAGEM-001/2020 strain from a patient in Turkey with confirmed COVID-19 [24]. The whole genomic sequence and replication characteristics of the hCoV-19/Turkey/ERAGEM-001/2020 strain was described. Here, we report the preclinical development of a whole-virion inactivated SARS-CoV-2 vaccine candidate (ERUCoV-VAC). The immunogenicity of inactivated vaccine candidate ERUCoV-VAC was evaluated in BALB/c, K18-hACE2, and ferret models. The protective efficacy of ERUCOV-VAC was determined in K18-hACE2 and ferrets and safety studies using the ferret model in preclinical studies.

## 2. Methods

### 2.1. Cell and Virus

Vero E6 cells (African green monkey kidney) obtained from ATCC (CRL 1586) were maintained in Dulbecco’s modified Eagle’s medium (DMEM) supplemented with 10% heat-inactivated fetal bovine serum (FBS) and 100 mM L-glutamine (Sigma–Aldrich, Darmstadt, Germany). The hCoV-19/Turkey/ERAGEM-001/2020 strain was isolated from a patient’s nasopharyngeal sample as described previously [27].

### 2.2. Facility and Ethics Statement

All the experiments with infectious SARS-CoV-2 were performed in a biosafety level 3 (BSL3)- and animal biosafety level 3 (ABSL3)-enhanced facility at ERAGEM. This study was approved by the Committee for Ethics on Animal Experiments (EUHADYEK/EU approval number 14/160) and the Committee for Animal Biosafety Level 3 Research (ERAGEM/EU protocol IP-3-14) of Erciyes University.

### 2.3. Animals

Six- to eight-week old and 12- to 14-month old female BALB/c mice were obtained from the Erciyes University Experimental Research and Application Center (DEKAM). K18-hACE2 (B6.Cg-Tg(K18-hACE2)2Prlmn/J transgenic mice) of 6 weeks old were purchased from the Jackson Laboratories (Bar Harbor, ME). The mice were maintained at 20–22 °C and a relative humidity of 50 ± 10% on a 12 h light/dark cycle, fed with rodent chow, and provided with tap water ad libitum. Male and female ferrets (Mustela Nivalis) that were 12 to 18 months old were purchased from Triple F Farms (Gillett, PA, USA). Animals were housed in an animal biosafety level 3 (ABSL3) enhanced facility at ERAGEM with a 12 h light/dark cycle and access to food and water in addition to environmental enrichment. Animals were monitored to ensure that they were eating, drinking, and behaving normally. Ferrets were monitored daily for care and health. Ferrets were lightly anaesthetized with ketamine (5 mg/kg), xylazine (0.5 mg/kg), and atropine (0.05 mg/kg) intramuscularly for immunization, collection of blood samples, nasal washes, and challenges with SARS-CoV-2. All animal care was conducted under the guidelines for animal experiments and performed as specified in regulation 5199, which describes animal protection and working with laboratory of animals in Turkey.

### 2.4. Virus Titration

The SARS-CoV-2 virus titre was determined by the tissue culture infective dose 50% (TCID50) method. Briefly, Vero E6 cells (0.4 × 10^6^ cells/mL) were seeded in 96-well plates and incubated for 18–24 h at 37 °C. Serial 10-fold dilutions of virus-containing samples were added to a 96-well culture plate and cultured for 5–7 days in a 5% CO2 incubator at 37 °C, and cells were observed for the cytopathic effect (CPE) under a microscope. The TCID50 was determined according to the Reed and the Muench methods [28].

### 2.5. Preparation of the Inactivated SARS-CoV-2 Vaccine

Vero E-6 cells were grown in a Nunc cell factory system (Thermo Fisher Scientific, Waltham, MA, USA) using DMEM containing 10% FBS. P4 viral stock was used to infect Vero E6 cells at a multiplicity of infection (MOI) 0.01. The supernatant was harvested at 72–96 h post-infection. The virus-containing supernatant was clarified by centrifugation at 3500 rpm for 20 min at 4 °C. The virus was inactivated with ß-propiolactone 1:1500 (*v*/*v*) at 4 °C and was further incubated at 37 °C for 2 h. Inactivation was confirmed by the inoculation of ß-propiolactone-treated samples on Vero E-6 cells. The inactivated virus was filtered using a 0.45 um filter (Millipore-sigma) following polyethylene glycol (PEG-8000, Promega, Madison, WI, USA) precipitation. Precipitated viral supernatants were treated with 20 U/mL of benzonase (Millipore; Burlington, MA, USA) overnight at 2–8 °C to digest host cell DNA. Column chromatography (Acta avant 150) was used for the purification following a tangential flow filtration system (Millipore cogent). After sterile filtration, an inactivated whole-virion SARS CoV-2 (ERUCoV-VAC) vaccine was formulated with aluminium hydroxide adjuvant (Alhydrogel, 250 µg per dose) (InvivoGen, San Diego, CA, USA). Different antigen concentrations (2.5 μg, 3 μg, 5 μg and 6 μg) were prepared in phosphate-buffered saline (PBS) and aluminium hydroxide adjuvant.

### 2.6. Analysis of Viral Antigen

The protein profiles of the vaccine antigen before and after purification were determined with sodium dodecyl sulfate polyacrylamide gel electrophoresis (SDS-PAGE) and Western blotting (WB) analysis as described previously [27].

### 2.7. Animal Studies

Six- to eight-week old female BALB/c mice and 12- to 14-month old female BALB/c mice were randomly divided into 3 groups (*n* = 6 per group). Mice in groups 1–2 were immunized on days 0 and 7 with a dose of 2.5 µg or 5 µg of ERUCoV-VAC by an intraperitoneal route. Mice in group 3 were similarly injected with PBS (served as normal controls). Serum samples were collected on 7, 14, 21, and 28 days after the injection for the evaluation of SARS-CoV-2-specific humoral response.

For SARS-CoV-2 challenge and immunogenicity experiments, K18-hACE2 mice were assigned to 3 experimental groups receiving either 3 µg (*n* = 13) or 6 µg (*n* = 10) of ERUCoV-VAC at days 0 and 21. The control group (*n* = 13) was administered with PBS. Serum samples were collected on days 7, 14, 21, 28, and 35 after the injection. Spleens were isolated from K18-hACE2 with the 3 µg dose group (*n* = 3) and the control group (*n* = 3) on day 35 for the ELISPOT assay. Six weeks post-initial immunization, all 3 groups of the K18-hACE2 mice were infected with 5 × 10^4^ TCID50 of SARS-CoV-2. Animals were monitored daily for signs of disease. Three animals for each group were euthanized 3 days following the challenge. Lungs, nasal turbinates, and brains were collected for virus isolation and virus load detection. The remaining animals were monitored twice daily for clinical signs of disease throughout the experiment. The mice that lost ≥25% of their initial body weight were humanely euthanized.

Ferrets were divided into 2 groups. Ferrets in group 1 (*n* = 6) were injected with PBS intramuscularly. Ferrets in group 2 (*n* = 6) received 6 µg of ERUCoV-VAC by intramuscular route. The ferrets were given booster injections at 3-week intervals. Serum samples were collected on days 14, 28, and 42 for evaluation of SARS-CoV-2-specific humoral response. Peripheral blood mononuclear cells (PBMCs) were isolated from the ferrets on day 48 for the ELISPOT assay. On day 49, animals were sedated and intranasally inoculated with 5 × 10^5^ TCID50 of SARS-CoV-2. After the viral challenge, nasal wash samples were collected on days 3, 7, and 14 for virus isolation and virus load detection.

### 2.8. RNA Extraction and SARS-CoV-2 Viral RNA Quantification by RT–qPCR

Nasal wash samples were collected in a 0.5 mL viral transport medium. Lungs, brains, and nasal turbinates were weighed and homogenized in 0.5 mL DMEM supplemented with 100 U/mL penicillin and 100 μg/mL streptomycin. The samples were centrifuged at 18,000× *g* for 10 min, and supernatants were collected. Then, RNA was extracted from 140 μL of the samples using the QIAamp viral RNA mini-kit (Qiagen). Detection of the SARS-CoV-2 virus was performed using Diagnovital (RTA Laboratories Inc, SARS-CoV-2 Real-Time PCR Kit v2.0, Istanbul, Turkey) on the Rotorgene Q thermal cycler platform (Qiagen) according to the manufacturer’s instructions. Ten-fold dilutions of SARS-CoV-2 RNA standards with known copy numbers were used to construct a standard curve.

### 2.9. Virus Isolation from Clinical Samples

The lungs, brains, and nasal turbinates were weighed and homogenized in 0.5 mL of DMEM supplemented with 100 U/mL penicillin and 100 μg/mL streptomycin. The samples were centrifuged at 18,000× *g* for 10 min, and supernatants were collected. The lungs, brains, nasal turbinates, and nasal wash samples were used for virus isolation. Vero-E6 cell monolayers in 24-well plates were inoculated with 100 uL of the organ supernatants and incubated for 1 h. The supernatant was removed and replaced with fresh DMEM containing 2% FBS and supplemented with 100 U/mL penicillin and 100 μg/mL streptomycin. The cytopathic effect was monitored daily. The culture supernatant from the wells showing CPE was confirmed by real-time RT–PCR.

### 2.10. ELISA

Commercial antigens were purchased from GenScript and comprised the nucleocapsid protein (N) (Z03488) and the S1/receptor-binding domain (S1-RBD) protein (Z03501). MaxiSorp ELISA plates (Nunc) were coated with SARS-CoV-2-specific antigens, whole inactivated antigen, or S1-RBD and N at concentrations of 10 μg/mL, 4 µg/mL, and 3 µg/mL, respectively, at 100 μL/well in carbonate buffer (0.1 M Na_2_CO_3_; 0.1 M NaHCO_3_; pH 9.4) overnight at 4 °C. Initial dilutions of the sera were 1/100 in two-fold serial dilutions. An ELISA plate was incubated at 37 °C for 1 h followed by washing with wash buffer (1X PBS + 0.05% Tween-20) four times. ELISA plates were incubated at 37 °C for 1 h with either a horseradish peroxidase (HRP)-conjugated polyclonal goat anti-mouse IgG (Southern Biotech) diluted 1:2000 or horseradish peroxidase (HRP)-conjugated polyclonal goat anti-ferret IgG (Abcam, cat no: ab112770) diluted 1:3000. The plate was washed again 4 times with wash buffer, and the ELISA was colourised with 100 uL of TMB substrate (Kementec) and placed into a dark space for 15–20 min. The plate was colourised with the peroxidase substrate solution, and the reaction was stopped by 2 M sulfuric acid. The absorbance was read at a wavelength of 450 nm (OD450) by a spectrophotometer (Biotek ELx80). The endpoint of the antibody titre was determined with a curve fit analysis of optical density (OD) values for serially diluted sera with a cut-off value set to three times the background signal. The results were recorded as the geometric mean titre (GMT) ± the standard error (S.E.).

### 2.11. Micro Neutralization Test (MNT)

ERUCoV-VAC-specific neutralizing antibody was identified using a microneutralization test (MNT) as described previously [29]. The titre of the neutralising antibody was determined as the highest dilution of serum at which the infectivity was neutralised in 50% of the cells in the wells. Seropositivity was defined as a titre ≥ 1/8.

### 2.12. ELISPOT Assay

ELISPOT assay was performed with mouse IFN-ELISpotPlus kit (Mabtech, Nacka Strand, Sweden) as previously described with minor modifications [30]. A total of 2.5 × 10^5^ splenocytes were stimulated at 37 °C for 18 h with MOI 0.1 of the SARS-CoV-2 or controls (splenocytes from PBS-inoculated K18-hACE2 mice); culture media alone (background control); concanavalin A 10 μg/mL (Sigma) (cell viability control). IFN-γ-secreting cells were revealed by adding streptavidin-alkaline phosphatase and 5-bromo-4-chloro-3′-indolyl phosphate/nitro blue tetrazolium-plus substrate. Spots were counted under a stereomicroscope (Leica). Whole blood samples from ferrets were collected in EDTA tubes and processed using leucosep (Grenier-bio). To each well, 3 × 10^5^ PBMCs were added and then stimulated at 37 °C for 24 h with MOI 0.1 of the SARS-CoV-2. The remaining steps of ELISPOT were performed as described above. Spot-forming units (SFUs) per million cells were represented after background subtraction from unstimulated cells.

### 2.13. Repeated Dose Toxicity

Repeated dose toxicity studies were performed under both national and international guidelines in compliance with OECD Principles of GLP. The study was carried out with the permission of the IDEA Local Ethics Committee of Experimental Animals. During the study, the Animal Welfare and Humanitarian Assessment Principles were followed under the EU Directives. Within the scope of the repeated dose toxicity test, an injection was made on days 0, 8, and 15. It was evaluated as a local tolerance test for 72 h after each injection. In the study, a total of 30 BALB/c mice (8-week-old, 15 females, and 15 males) and 9 ferrets (5 males and 4 females) were tested. Animals (BALB/c mice, ferret-Mustela Nivalis) were administered (per vaccine dose: 0.5 mL; total vaccine dose: 6 micrograms) via an intramuscular route with ERUCoV-VAC on days 0, 8, and 15. During the test, all animals were weighed regularly, and their feed consumption was observed. All animals were observed for mortality and clinical signs during the experimental period. The animals were checked daily in terms of their physiological interest in the environment in terms of health parameters, general condition, feather integrity, stool forms, feed and water consumption, and cage cleaning. The weight measurements of the animals were performed periodically, and weekly weight differences were checked. Blood samples were collected under anaesthesia (xylazine-ketamine), and clinical evaluations, such as haematology and serum chemistry, using the validated biochemistry analysis method were performed. Samples for haematology and clinical biochemistry were collected on days 2 and 21 for the main groups and day 28 for the recovery groups. Animals were euthanized either on day 21 (main groups) and/or on day 28 (recovery groups), after blood sampling. Mice and ferrets were necropsied and observed macroscopically. Organs such as the brain, thymus, spleen, ovaries, uterus, heart, kidneys, testes, liver, adrenals, lungs, epididymides, and prostate with seminal vesicles and coagulating glands were weighed, and all organs were collected for microscopic examinations as per the WHO guidelines. Organs for microscopic examination were preserved in 10% neutral buffered formalin (NBF). Tissues were processed and stained with haematoxylin and eosin.

### 2.14. Statistical Analysis

The graphic drawing and data analysis were performed using GraphPad Prism 7.0. The Kaplan–Meier survival curve with the log-rank (Mantel–Cox) test was applied to show the survival percentage of mice. The ordinary one-way ANOVA was used to compare groups in the viral copy number and TCID_50_/mL. For ELISA data analysis, two-way ANOVA was used for the comparison between groups. Comparison between different groups from the neutralizing antibody assay and ELISPOT was performed using a two-sided Mann–Whitney test. Two statistical methods were used for the ELISPOT data analysis, the unpaired *t*-test, and the Mann–Whitney U test. An unpaired *t*-test was used for the normal data distribution. Unlike this, the Mann–Whitney U test was used for the non-normal data distribution. To determine the significant differences between groups *p* values less than 0.05 were considered to be statistically significant where *** denotes 0.001; ** denotes 0.01; and * denotes 0.05. Error bars represent mean ± standard deviation.

## 3. Results

### 3.1. Generation of ERUCoV-VAC as a Vaccine Platform

**Figure 1:**
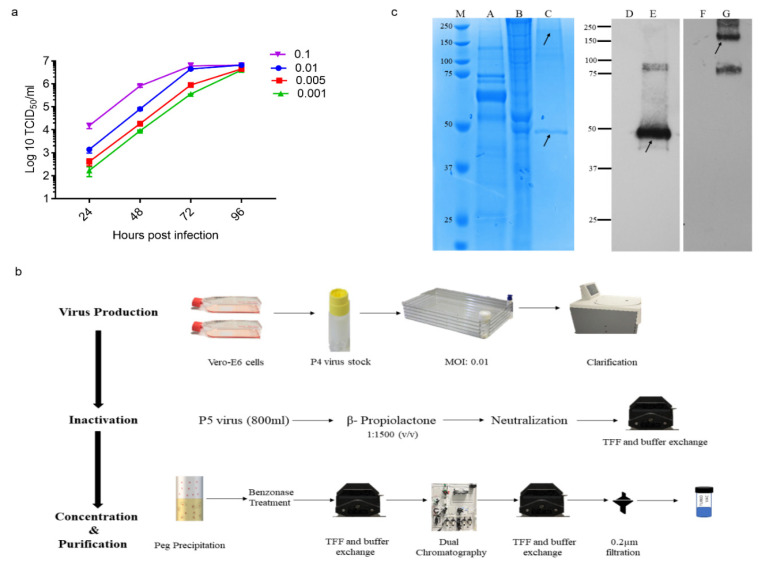
Preparation of ERUCoV-VAC. (**a**) The viral kinetics of the hCoV-19/Turkey/ERAGEM-001/2020 strain at different multiplicities of infection (MOI). (**b**) Flowchart of ERUCoV-VAC preparation. (**c**) Protein profiles of the hCoV-19/Turkey/ERAGEM-001/2020 virus strain before and after purification. Total proteins were separated by Nu-page 10% bis-tris SDS-PAGE gel. Lane 1, protein molecular weight marker (M). Lane A, uninfected Vero E-6 cells as a negative control. Lane B, Vero E-6 cells infected with the hCoV-19/Turkey/ERAGEM-001/2020 virus strain. Lane C, purified hCoV-19/Turkey/ERAGEM-001/2020 virus strain. Western blot analysis of purified hCoV-19/Turkey/ERAGEM-001/2020 virus strain probed with a human antibody to the SARS-CoV-2 nucleocapsid protein (1:2500) (GenScript; HC2003) (Lane E) or a rabbit polyclonal to SARS-CoV-2 spike glycoprotein (lane G). Uninfected cell lysates probed with the SARS-CoV-2 nucleocapsid protein (Lane D) and a rabbit polyclonal to SARS-CoV-2 spike glycoprotein (F) were used as a negative control. The arrows indicate that the bands at approximately 180 kDa and 48 kDa represent spike glycoprotein and nucleocapsid protein, respectively.

We previously reported the isolation of the hCoV-19/Turkey/ERAGEM-001/2020 strain from a patient in Turkey with confirmed COVID-19. The hCoV-19/Turkey/ERAGEM-001/2020 strain was closely related to the Wuhan Hu-1 strain but had six more variants [27]. The genetic stability of the hCoV-19/Turkey/ERAGEM-001/2020 strain was assessed by 10 generations in Vero E-6 cells, and its whole-genome P4 and P10 stocks were sequenced by next-generation sequencing. Compared with P4, we found one synonymous mutation to correspond to the genomic position T22213C (S gene) in P10, suggesting that the hCoV-19/Turkey/ERAGEM-001/2020 strain was genetically stable (Table S1). The inoculation of P4 stock with different multiplicities of infection (MOI), 0.001, 0.005, 0.01, and 0.1, compared to Vero E6 cells resulted in 6–6.5 log10 TCID50/mL between 3 and 4 dpi (Figure 1a). To generate research-grade ERUCoV-VAC production, Vero E6 cells were cultured in a multi-tray cell factory system (Thermo Fisher Scientific, Waltham, MA, USA). Then, P4 virus stock was amplified in Vero E6 cells at an MOI of 0.01, and the supernatant was harvested at 72–96 h post-infection by centrifugation at 3500 rpm for 20 min at 4 °C. The virus was inactivated with ß-propiolactone (Invitrogen) (1:1500 (*v*/*v*)) at 4 °C for 24 h and was further incubated at 37 °C for 2 h. Inactivation was confirmed by the inoculation of ß-propiolactone-treated samples on Vero E-6 cells. No cytopathic effect was observed on Vero E6 cells inoculated with the inactivated virus. The inactivated supernatant (800 mL) was concentrated and partially purified by tangential flow microfiltration following polyethylene glycol (PEG) precipitation and further purified with a dual chromatography system (Figure 1b). The purity of ERUCoV-VAC was assessed by total protein staining after separation by SDS-PAGE. Inactivated and purified ERUCoV-VAC was recognized by anti-spike and anti-N protein antibodies in the Western blot (Figure 1c).

A total of 30 BALB/c mice (15 females and 15 males) and 9 ferrets (5 males and 4 females) were used for the safety evaluation of ERUCoV-VAC. Repeated injections of (N + 1 dose regimen) a high dose of an adjuvanted formulation of ERUCoV-VAC (6 μg per dose) was administered in BALB/c mice and ferrets. The general condition of the animals, feed consumption, and water intake were recorded. No abnormal situation at the injection site was observed in the examinations of oedema, swelling, sensitivity to touching the area, scab, wound, and hair integrity in all groups. We did not observe any signs of illness, fever, weight loss, or stress in the animals (Figure S1). The animals were euthanized either on day 21 (main groups) and/or on day 28 (recovery groups), after blood sampling, and were necropsied. Histopathological examination of organs such as the livers, lungs, spleens, and kidneys of all animals administered with adjuvanted vaccine formulations was normal (Figure S2).

### 3.2. ERUCoV-VAC Induces Humoral Immune Responses in Old and Young BALB/c Mice

**Figure 2:**
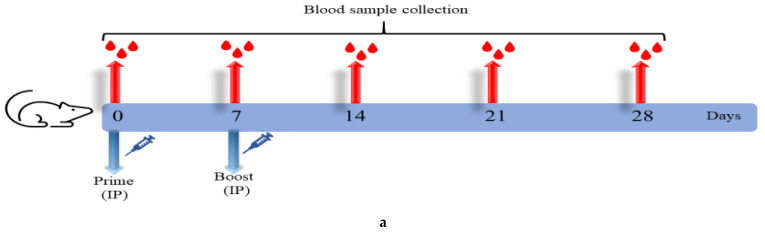

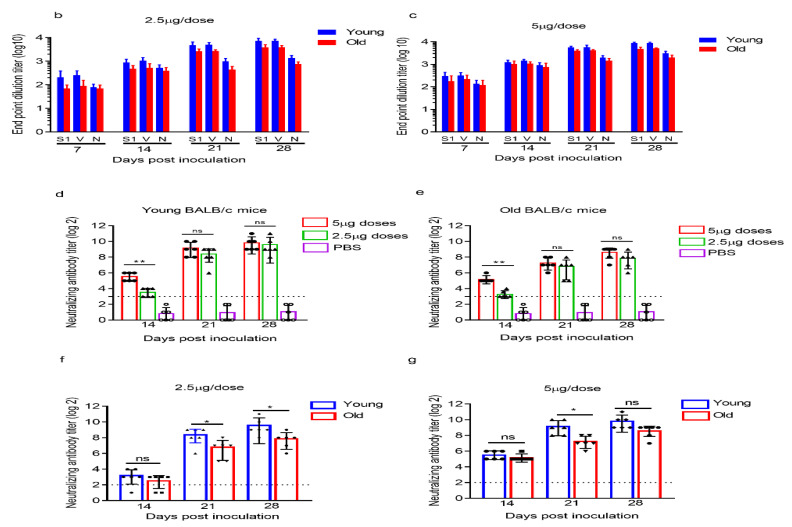
ERUCoV-VAC immunogenicity in old and young BALB/c mice. (**a**) Schematic diagram of sample collection and immunization regimens. Serum IgG titres were detected by SARS CoV-2-specific S1-RBD (S1), virion (V), and nucleocapsid (N) ELISA in young (*n* = 6) and old BALB/c (*n* = 6) mice vaccinated with 2.5 μg (**b**) and 5 μg (**c**) doses of ERUCoV-VAC. Serum Nab titres determined by microneutralizing assay in young (**d**) and old mice (**e**) vaccinated with 2.5 μg and 5 μg doses of ERUCoV-VAC. Comparison of serum Nab titres in young and old BALB/c mice vaccinated with 2.5 μg (**f**) and 5 μg (**g**) doses of ERUCoV-VAC. The statistical significance was assessed using a two-way ANOVA test and a Mann–Whitney test between groups; *p* values less than 0.01 were considered to be statistically significant where ** denotes 0.01; and * denotes 0.05; ns: not significant. Error bars represent mean ± standard deviation. The dotted line illustrates the highest value measured in the normal control group.

Immunogenicity of the ERUCoV-VAC was tested in the young and old BALB/c mice vaccinated intraperitoneally either low dose (2.5 μg) or high dose (5 μg) or control group (PBS), each comprising six mice. Priming and boosting were performed on days 0 and 7, respectively (Figure 2a). The levels of anti-SARS-CoV-2 IgG responses against virion-based S1-RBD protein and N protein at days 7, 14, 21, and 28 after immunization were evaluated (Figure 2b,c). The seroconversion rate in the young and old BALB/c mice reached 100% at 14 days after immunization in high- and low-dose groups. There was a strong increase in the virion and S1-RBD-based IgG responses from day 14 to 28 in the vaccinated groups. Notably, S1-RBD-specific IgG levels were similar to virion-based IgG levels and higher than those of antibodies targeting nucleocapsid protein in immunized mice, suggesting that the SARS-CoV-2 S1-RBD domain is more immunogenic than the nucleocapsid domain (Figure 2b,c).

To evaluate if the vaccine-elicited antibodies were capable of neutralizing SARS-CoV-2, the immunized mice sera were analysed by MNT in Vero E6 cells. Although the neutralizing antibody (Nab) response was detected in both immunization groups at 14 days after immunization, the geometric mean Nab titres of the high-dose group were significantly higher than those of low-dose group (Figure 2d,e). At days 21 and 28 after vaccination, the high-dose group exhibited a stronger response compared to the low-dose group in vaccinated young and old BALB/c mice groups (although not statistically significant) (Figure 2d,e). However, the mean Nab titres observed in the young mice were significantly higher (*p* < 0.001) than those observed in old mice at days 21 and 28 after vaccination (Figure 2f,g). Taken together, although old BALB/c mice had lower antibody levels than young BALB/c mice, ERUCoV-VAC is highly immunogenic and elicited an immune response in both old and young BALB/c mice.

### 3.3. ERUCoV-VAC Protects K18-hACE2 Transgenic Mice against a Lethal SARS-CoV-2 Challenge

**Figure 3:**
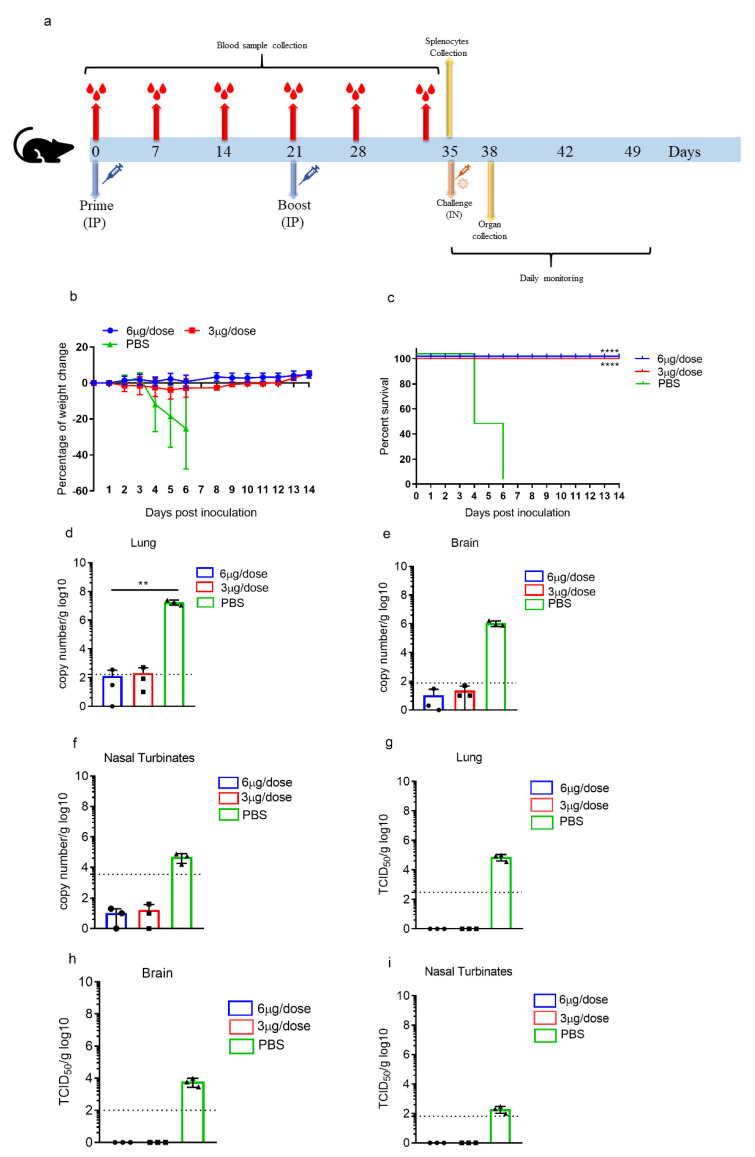
Protective efficacy of ERUCoV-VAC in K18-hACE2 transgenic mice. (**a**) Scheme of sample collection, immunization regimens, and SARS CoV-2 challenge. Groups of K18-hACE2 transgenic mice immunized on days 0 and 21 with doses of 3 µg (*n* = 13) or 6 µg (*n* = 10) of ERUCoV-VAC or with a placebo (*n* = 13) via the intraperitoneal route. The K18-hACE2 mice (*n* = 10 per group) were challenged at 2 weeks after the second immunization with 5 × 10^4^ TCID50 of SARS-CoV-2 in a volume of 60 µL by the intranasal route. Bodyweight (**b**) and survival (**c**) were evaluated according to the indicated timeline. The K18-hACE2 transgenic mice that lost ≥ 25% of their initial body weight were humanely euthanized. On day 3 after the challenge, three K18-hACE2 transgenic mice in each group were euthanized, and lungs, brains, and nasal turbinates were collected for qPCR and virus titration via TCID50 assay. qPCR was performed by targeting viral N gene for lungs (**d**), brains (**e**), and nasal turbinate (**f**) using a Diagnovital real-time PCR kit. A standard curve was generated using a viral RNA. Ten-fold dilutions of viral RNA were prepared, and negative control samples were included in each assay. The Ct value for each sample was converted into log_10_ viral copies/g tissue according to the standard curve. The dotted line indicates the highest value measured in the normal control group, which were 2.16 log10, 1.75 log10, and 3.35 log10 N copy number/g in the lungs, brains, and nasal turbinates, respectively. Live virus titres of the lungs (**g**), brains (**h**), and nasal turbinates (**i**) were determined via TCID50 assay at day 3 post-challenge. The statistical significance was assessed using a two-way ANOVA test, and *p* values less than 0.01 were considered to be statistically significant where ** denotes 0.01. Error bars represent mean ± standard deviation.

In order to evaluate the protective efficacy of ERUCoV-VAC, K18-hACE2 mice were assigned to three experimental groups receiving either 3 µg (*n* = 13) or 6 µg (*n* = 10) of ERUCoV-VAC at days 0 and 21. The control group (*n* = 13) was administered with PBS. On day 14 after the second immunization, the K18-hACE2 mice were intranasally challenged with 5 × 10^4^ TCID50 of SARS-CoV-2 (Figure 3a).

The K18-hACE2 mice vaccinated with either 3 μg or 6 μg doses of ERUCoV-VAC were fully protected after challenge, and there were no obvious weight loss changes among the two vaccinated groups, whereas mice vaccinated with PBS succumbed to infection within 6 days due to ≥25% weight loss or a poor body condition (Figure 3b,c). Three animals for each group were euthanized three days following the challenge. Lungs, nasal turbinates, and brains were collected for virus isolation and virus load detection. Viral RNA was detected in all three unvaccinated animals. The highest viral load was found in lung tissue (~10^7^ RNA copies equivalents per gram) compared to the brain (~10^6^ RNA copies equivalents per gram) and nasal turbinates (~10^4^ RNA copies equivalents per gram) (Figure 3d–f). Viral RNA in lung tissue was detectable at very low levels in two vaccinated animals, one of which was in the 3 μg dose group, and the other one was in the 6 μg dose group (Figure 3d). No viral RNA could be detected in nasal turbinates and brain tissues obtained from the vaccinated animals (Figure 3e,f). We were unable to find the infectious virus in the tissues of ERUCoV-VAC-vaccinated animals (Figure 3g–i). By contrast, the unvaccinated control animals showed high titres of replicating virus in lungs, brain tissues, and nasal turbinates: 4.8 log10 TCID50/g, 3.9 log10 TCID50/g, and 2.1 log10 TCID50/g, respectively (Figure 3g–i). Even though some vaccinated animals showed a low level of viral RNA, no detectable infectious virus was found in the lungs, nasal turbinates, or brains of ERUCoV-VAC-vaccinated animals.

**Figure 4:**
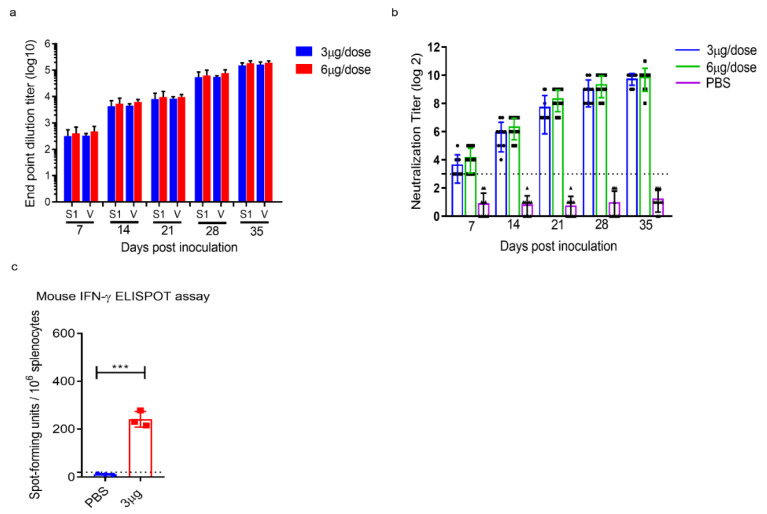
ERUCoV-VAC immunogenicity in K18-hACE2 transgenic mice. (**a**) Serum IgG titres were detected by SARS CoV-2 specific S1-RBD (S1) and virion (V) ELISA in vaccinated with 3 μg and 6 μg doses of ERUCoV-VAC in K18-hACE2 transgenic mice. (**b**) Serum Nab titres determined by microneutralizing assay in K18-hACE2 transgenic mice vaccinated with 3 μg and 6 μg doses of ERUCoV-VAC. (**c**) Cellular immune responses in 3 μg dose group (*n* = 3) and control group (*n* = 3) were analysed at day 35 following first immunization by an IFN-γ-based ELISPOT assay. The statistical significance was assessed using a two-way ANOVA test and unpaired *t*-test (ELISPOT); *p* values less than 0.01 were considered to be statistically significant significant where *** denotes 0.001; ns: not significant. Error bars represent mean ± standard deviation. The dotted line indicates the highest value measured in the normal control group.

We detected the S1-RBD and virion antibody titres in all vaccinated K18-hACE2 one week after the first immunization. After a booster dose, antibody titres in both immunization groups increased gradually (Figure 4a). The neutralizing antibodies in the vaccinated animals were detectable at week 2 after the first immunization. As expected, the neutralizing antibody response markedly increased in both immunization groups after the second immunization (Figure 4b).

Next, we evaluated the T-cell responses in K18-hACE2 mice immunized with 3 μg of ERUCoV-VAC. On day 35 post-immunization, SARS CoV-2-specific T cells were re-stimulated with the live virus in vitro and analysed for secreting IFN-gamma by enzyme-linked immunospot (ELISPOT). The results demonstrated that T cells secreting gamma interferon (IFNγ) from K18-hACE2 mice immunized with ERUCoV-VAC had higher IFN-γ responses than the control group (Figure 4c).

Collectively, these data show that two doses of ERUCoV-VAC-induced humoral and cellular immune responses led to the protection of K18-hACE2 mice from a lethal SARS-CoV-2 challenge.

### 3.4. ERUCoV-VAC Reduces Upper Respiratory Tract SARS-CoV-2 Infection in Ferrets

**Figure 5:**
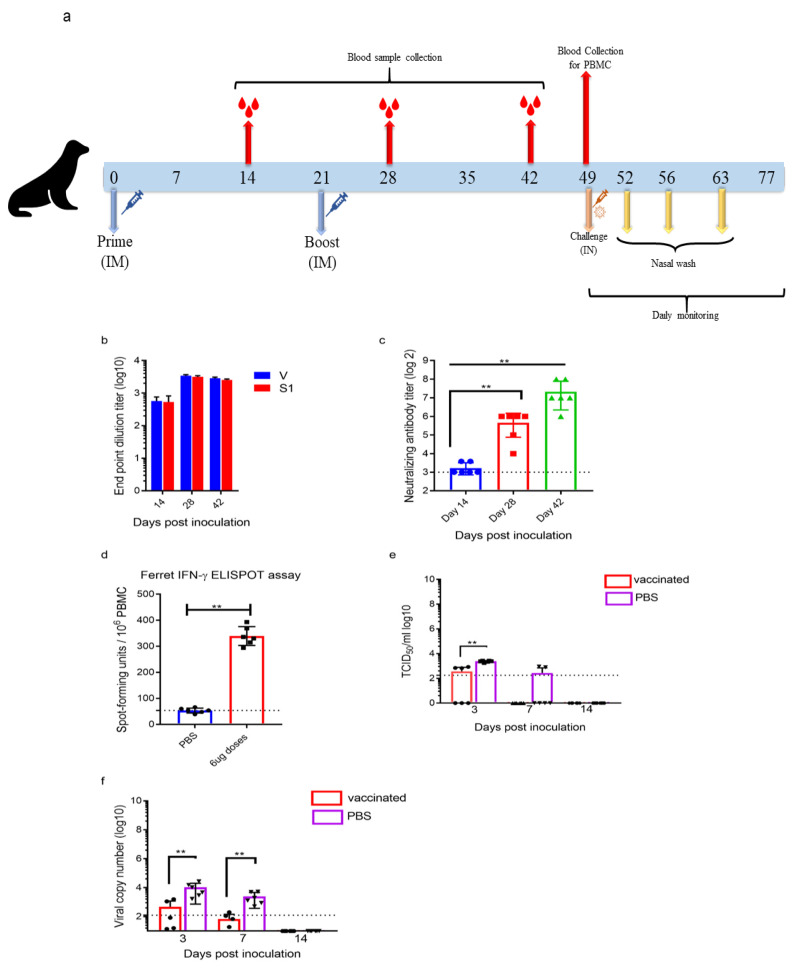
Immune responses and protective efficacy of ERUCoV-VAC in ferrets. (**a**) Scheme of sample collection, immunization regimens, and SARS CoV-2 challenge. Groups of ferrets (*n* = 6) immunized on days 0 and 21 with 6 µg of ERUCoV-VAC or with a PBS (*n* = 6) via the intramuscular route. The ferrets were challenged 4 weeks after the second immunization with 5 × 10^5^ TCID50 of SARS-CoV-2 by the intranasal route. (**b**) Serum IgG titres were detected by SARS CoV-2-specific S1-RBD (S1) and virion (V) ELISA in ferrets vaccinated with a 6 μg dose of ERUCoV-VAC. (**c**) Serum Nab titres determined by microneutralizing assay in ferrets vaccinated with a 6 μg dose of ERUCoV-VAC. (**d**) Cellular immune responses in the 6 μg dose group (*n* = 6) and control group (*n* = 6) were analysed on day 28 following the second immunization by an IFN-γ-based ELISPOT assay. On day 28 after the second immunization, a total of 12 ferrets, both vaccinated (*n* = 6) and unvaccinated (*n* = 6), were intranasally challenged with 5 × 10^5^ TCID50 of SARS-CoV-2. Live virus titres (**e**) and viral load of SARS CoV-2 (**f**) in nasal washes obtained from ferrets according to the indicated timeline after challenge. The dotted line indicates the highest value measured in the normal control group, which was 2.125 log_10_ N copy number/mL in the nasal washes. The statistical significance was assessed using a Mann–Whitney test; *p* values less than 0.01 were considered to be statistically significant where ** denotes 0.01; ns: not significant. Error bars represent mean ± standard deviation.

Ferrets are naturally susceptible to human respiratory viruses and have been used as a model for diseases caused by the influenza virus, the respiratory syncytial virus, and the Nipah virus [31]. Recently, some studies have demonstrated that ferrets are a suitable mammalian model for SARS CoV-2, which efficiently replicates in its upper respiratory tracts [32,33]. To examine whether the ERUCoV-VAC can induce protective immunity in the upper respiratory tracts of ferrets, the animals were divided into two groups. Ferrets in group 1 (*n* = 6) were injected with PBS intramuscularly. Ferrets in group 2 (*n* = 6) received 6 µg of ERUCoV-VAC via the intramuscular route. The ferrets were given booster injections at 3-week intervals (Figure 5a). The ability of ERUCoV-VAC to induce antibody and T-cell responses was analysed by ELISA, microneutralization, and IFN-γ ELISPOT assay. As shown in Figure 5b,c, all vaccinated ferrets produced S1-RBD- and virion-specific serum IgG antibodies and NAbs at week 4 after the second immunization. SARS-CoV-2-specific cellular responses were assessed in vaccinated animals by IFN-γ ELISPOT. The IFNγ response of T cells in ferrets inoculated with ERUCoV-VAC was significantly higher than that of inoculated ferrets in the PBS-immunized group (Figure 5d). These ferrets were challenged intranasally with 5 × 10^5^ TCID50 of SARS-CoV-2 at week 4 after the second immunization. The animals did not show any symptoms, except that some ferrets in the control group displayed reduced activity. No weight changes were observed for all ferrets throughout the time course. Nasal washes were taken at days 3, 7, and 14 dpi for TCID50 assays and viral load analysis of SARS-CoV-2 by qPCR. Furthermore, three out of six vaccinated ferrets showed viral titres via TCID50 at 3 dpi (Figure 5e). No virus titres were obtained in the nasal washes from the vaccinated animals via TCID50 at 7 and 14 dpi (Figure 5e). Even though all vaccinated ferrets showed SARS CoV-2 RNA at day 3 post-challenge, a significant reduction in SARS CoV-2 RNA levels was determined at 7 dpi (Figure 5f). By contrast, viral titres were obtained via TCID50 from all the infected control ferrets at day 3 dpi (Figure 5e). Additionally, two out of six infected control ferrets showed viral titres at day 7 dpi, even though all animals had high SARS CoV-2 RNA levels (Figure 5e,f). At day 14 dpi, no infectious virus or SARS CoV-2 RNA was found in any of the infected control ferrets (Figure 5e,f). Taken together, the rate of viral clearance in the upper respiratory tracts of ferrets immunized with ERUCoV-VAC was much greater than that of control animals, suggesting that ERUCoV-VAC provided immune protection in the ferrets.

## 4. Discussion

The development of a safe and effective vaccine to protect against COVID-19 is a global health priority due to the current high rate of disease transmission and the high number of hospitalizations and deaths that threaten to overwhelm health systems in many countries [18]. The first genome sequence of SARS-CoV-2 was published on 11 January 2020, stimulating outstanding efforts in the development of various vaccine candidates against the disease [34]. According to WHO, on 1 October 2021, there were 123 candidate vaccines in human clinical trials and 194 candidates in preclinical development worldwide (https://covid19.who.int, accessed 4 October 2021). Several vaccines have subsequently been granted emergency authorization for use in humans, which represents a major milestone in the fight against the COVID-19 pandemic [35]. Most of these vaccines are based on viral vectors (e.g., adenovirus, University of Oxford/AstraZeneca, Gamaleya Sputnik V, and Johnson & Johnson), mRNA (Moderna and Pfizer-BioNTech), or whole inactivated (Sinovac Biotech and Sinopharm) vaccines. However, worldwide access to these vaccines is limited, particularly in low-income or developing countries, due to extreme cold-chain requirements, high costs, and an insufficient supply of the SARS CoV-2 vaccines [36,37]. Therefore, additional SARS-CoV-2 vaccines are needed to meet the global demand. Most of the current SARS-CoV-2 vaccines are based on the spike (S) protein to elicit an immune response against SARS-CoV-2 [7,38,39]. However, mutations in the S protein lead to new variants of SARS-CoV-2 that become dominant worldwide, including B.1.1.7 (Alpha), B.1.351 (Beta), P.1 (Gamma), and B.1.617.2 (Delta) lineages (www.who.int, accessed on 22 June 2021), and they have created serious concerns about the reduction in the vaccine efficacy [40,41,42]. An advantage of inactivated vaccines over the current SARS-CoV-2 vaccines is that the immune responses to a SARS-CoV-2-inactivated vaccine would target not only the spike protein (S) of SARS-CoV-2 but also other viral proteins, including the matrix (M), envelope (E), and nucleocapsid (N). An advantage of inactivated vaccines over the current SARS-CoV-2 vaccines is that the immune responses to a SARS-CoV-2-inactivated vaccine would target not only the spike protein (S) of SARS-CoV-2 but also other viral proteins, including the matrix (M), envelope (E), and nucleocapsid (N). Although this ensures a broader response, it is also important for considering the risks of antibody-dependent enhancement (ADE) of disease caused by coronaviruses. Regarding the mechanism of the ADE, immune complexes formed between the virus and non-neutralising antibodies, and poorly neutralising antibodies bind to receptor molecules called Fcy receptors (FcyRs), which are expressed broadly on monocytes and macrophages. This interaction leads to the internalization of the virus particle to enter the cell [43]. SARS-CoV vaccination studies in animal models have produced widely varying results in terms of ADE and immunopathology. It has been shown that antibodies produced by proteins such as nucleocapsid trigger the production of non-neutralising antibodies that could favour the ADE mechanism, which was also observed for SARS-CoV and MERS-CoV in animal models [44,45]. Wang et al. demonstrated that ADE is mainly induced by diluted antibodies against spike proteins rather than nucleocapsid protein [46]. In contrast, Luo et al. showed that rhesus macaques vaccinated with an inactivated SARS-CoV vaccine induced a low titre of neutralising antibodies and did not show higher levels of lung pathology when compared to placebo controls [47]. A recent study reported that the Chinese rhesus monkey was used as an animal model to assess the relationship between ADE and the neutralising antibody titre induced by the SARS vaccine, which encodes the complete SARS-CoV viral spike poxvirus vector. They found a positive correlation between the amount of neutralising antibody in serum and the degree of pathological injury in the lungs [48]. Based on observations, it is reasonable to consider that SARS-CoV-2 vaccines may cause ADE. Although SARS-CoV-2 vaccines have not been associated with antibody enhancement disease in either preclinical or clinical studies, we cannot ignore the ADE risk for SARS-CoV-2 antibodies [49,50].

Inactivated vaccines against SARS CoV-2, such as BBIBP-CorV (Sinopharm Beijing), inactivated vaccineWIBP (Sinopharm Wuhan), Coronavac (Sinovac), and BBV152 (Bharat Biotech), have been approved for emergency use in several nations [18,51]. Moreover, inactivated vaccines have been widely used for the prevention of viral diseases, such as polio, influenza, hepatitis A, and rabies [52]. The safety profile and effectiveness of well-characterised Vero cell-based inactivated vaccines make this an attractive platform for rapid vaccine development and the deployment of COVID-19 [20,21,52,53,54]. Here, we present the preclinical immunogenicity, protective efficacy, and safety evaluation of an inactivated SARS-CoV-2 vaccine candidate (ERUCoV-VAC) in three animal models, BALB/c mice, transgenic mice (K18-hACE2), and ferrets.

ERUCoV-VAC was formulated with aluminium hydroxide, which is the most widely used vaccine adjuvant, with extensive safety records over the decades [53,55,56]. In this study, the safety evaluation of ERUCoV-VAC formulated in aluminium hydroxide showed no local or systemic toxic manifestations in mice and ferrets, as demonstrated by the repeated dose toxicity with no changes in body weight or body temperature (Figure S1). The aluminium adjuvant formulations were found to develop high titres of neutralizing antibodies by mechanisms that are obscured. Reports propose that they benefit the antibody reaction by favouring the activation and trafficking of antigen-presenting cells to lymphoid tissues in addition to triggering the inflammasome and complementing activation [57]. In general, the immune response elicited from aluminium hydroxide is primarily Th2-biased with the induction of strong humoral responses via NAbs [58,59]. Neutralizing antibodies play a critical role in protection against SARS-CoV-2 infection and have been used as an immune correlate of protection in assessing vaccine efficacy [59,60,61]. Inactivated SARS-CoV-2 vaccine candidates have been shown to induce high levels of antigen binding and NAb titres in preclinical studies [19,62,63]. In this study, we first analysed the immunogenicity of the ERUCoV-VAC in young and old BALB/c mice. Although the old BALB/c mice had lower antigen-binding and neutralizing antibody levels than those of young BALB/c mice, ERUCoV-VAC is highly immunogenic and elicited an immune response in both old and young BALB/c mice (Figure 2). In this study, a strong neutralising antibody was also generated in all immunised K18-hACE2 mice (512–2048) and also in the ferrets (128–1024) (Figure 4b and Figure 5c).

The protective efficacy of inactivated vaccine candidates has been evaluated in hamsters and non-human primates as larger animal studies contribute to a more reliable recognition of the immune responses in humans [64,65]. After challenges with virulent SARS CoV-2 in the vaccinated animals, studies revealed that it did not result in death but led to weight loss, symptomatic disease, and viral replication in various tissues [18,21,51]. In this study, K18-hACE2 mice were chosen to test the vaccine efficacy due to the existing evidence on its contribution to severe disease and lethality [25,26]. Our data demonstrated that two doses of either 3 μg or 6 μg of the ERUCoV-VAC elicited strong immune responses and fully protected K18-hACE2 mice from morbidity and mortality, whereas mice vaccinated with PBS succumbed to infection within 6 days (Figure 3c). Additionally, we used the ferret model to assess the safety and efficacy of ERUCoV-VAC. Ferrets are suitable animal models for respiratory viruses such as influenza and respiratory syncytial virus (RSV), as they have anatomical and physiological features of the respiratory tract that are similar to humans [31]. SARS-CoV-2 infection exhibits a mild clinical disease in ferrets [23]. Several studies clearly showed that both virus recovery and the highest viral RNA levels were detected from the nasal turbinates, indicating the main site for viral replication and transmission in the upper respiratory tract [66,67,68,69]. Therefore, ferrets provide a useful animal model for studying viral transmission and the human upper respiratory diseases. In this study, we evaluated whether the ERUCoV-VAC can induce protective immunity in the upper respiratory tracts of ferrets. Our results demonstrate that compared to non-vaccinated ferrets, a significant reduction in SARS CoV-2 RNA levels in the nasal washes was found, indicating that ERUCoV-VAC provided immune protection to the ferrets. (Figure 5f).

Inactivated vaccines are mostly associated with stimulating B lymphocytes to produce antibodies. T-cell responses generated by inactivated vaccines are weak and much less well characterized than antibody-mediated immunity [70]. In this study, the cellular immune response elicited by ERUCoV-VAC in K18-hACE2 mice and ferrets was measured by ELISPOT assay. Notably, immunization with ERUCoV-VAC was capable of eliciting higher numbers of IFN-γ SFCs compared with the numbers observed for the non-vaccinated K18-hACE2 mice and ferrets (Figure 4c and Figure 5d). The results of our present study were in agreement with the previous reports that inactivated vaccines against SARS CoV-2 could induce IFN-γ responses. Ganneru et al. demonstrated that inactivated SARS-CoV-2 vaccine (BBV152) showed elevated levels of IFN-γ producing CD4+ cell population [63]. One previous study revealed that the inactivated vaccine (CoronaVac) induces low levels of IFN-γ responses in participants [71]. It has been showed that inactivated COVID-19 vaccine (BBIBP-CorV) induced T-cell responses to multiple structural proteins (S, N, and E proteins) of SARS-CoV-2 [72]. Altogether, inactivated vaccines against SARS-CoV-2 may induce T-cell responses in addition to humoral responses, and cellular responses may be involved in the protection provided by inactivated vaccines.

Hence, the vaccine was successful in demonstrating its safety profile for extensive use in humans. There are some limitations to our study. One of the safety concerns about the application of aluminium adjuvants stem from the fact that Th2-type immune responses might favour vaccine-enhanced respiratory disease (VAERD) [16]. However, studies based on these aluminium-adjuvanted coronavirus vaccines state no such lines of evidence [20,63]. Rather than exaggerating the disease, the aluminium formulations were instead observed to decrease the immunopathology when compared with other unadjuvanted coronavirus vaccines [73]. These shortcomings, however, warrant in-depth review to eliminate false interpretations. Another disadvantage is that we did not detect the lung pathology or immunohistochemistry in the animal models after infection with SARS COV-2. However, in spite of this limitation, we showed that three K18-hACE2 mice from the unvaccinated group showed a high viral load in their lungs, brains, and nasal turbinates (~10^7^ RNA, ~10^6^ RNA, and ~10^4^ RNA copy equivalents per gram, respectively) at 3 dpi (Figure 3d–f). We did not find any viral load in brain tissues or nasal turbinates from the vaccinated groups, but we found viral RNA at very low levels in the lung tissues of two vaccinated animals (Figure 3d–f), suggesting that ERUCoV-VAC restrained the virus replication in the lower respiratory tracts in K18-hACE2 mice. However, ERUCoV-VAC is yet to be assessed for further long-term protective efficacy and the cross-reactive neutralization of other SARS-CoV-2 variants.

## 5. Conclusions

We presented the preclinical immunogenicity, protective efficacy, and safety evaluation of a whole-virion inactivated SARS-CoV-2 vaccine candidate (ERUCoV-VAC) formulated in aluminium hydroxide in three animal models: BALB/c mice, transgenic mice (K18-hACE2), and ferrets. ERUCoV-VAC was highly immunogenic and elicited a strong immune response in BALB/c mice. In our findings, the protective efficacy of the vaccine in K18-hACE2 showed that ERUCoV-VAC induced complete protection of the mice from a lethal SARS-CoV-2 challenge. Similar viral clearance rates with the safety evaluation of the vaccine in upper respiratory tracts were also positively appreciable in the ferret models. Based on the preclinical data presented here, ERUCoV-VAC has been authorized by the Turkish Medicines and Medical Devices Agency and has now entered phase 3 clinical development (NCT04942405). The name of ERUCoV-VAC has been changed to TURKOVAC in the phase 3 clinical trial.

## Data Availability

The data that support the findings of this study are available on request from the corresponding author.

## Supplementary Materials

The following are available online at https://www.mdpi.com/article/10.3390/vaccines9111266/s1, Figure S1. Body temperature and body weight of ferrets administered N + 1 (6 μg Ag and Alhydrogel) dose regimen. Figure S2. Representative Hematoxylin and Eosin-stained liver, lung, kidney, and spleen tissues from ferrets administered with saline buffer and adjuvanted vaccine (6 μg Ag and Alhydrogel). Table S1: List of detected variants in hCoV-19/Turkey/ERAGEM-001/2020 strain. Table S2: List of materials used in this study.

## References

[1] Masters P.S., Perlman S., Knipe D.M., Howley P.M. (2013). Coronaviridae. Fields Virology.

[2] Sola I., Almazan F., Zuniga S., Enjuanes L. (2015). Continuous and discontinuous RNA synthesis in coronaviruses. Ann. Rev. Virol..

[3] Ozdarendeli A., Ku S., Rochat S., Williams G.D., Senanayake S.D., Brian D.A. (2001). Downstream sequences influence the choice between a naturally occurring noncanonical and closely positioned upstream canonical heptameric fusion motif during bovine coronavirus subgenomic mRNA synthesis. J. Virol..

[4] Wu H.-Y., Ozdarendeli A., Brian D.A. (2006). Bovine coronavirus 5 -proximal genomic acceptor hotspot for discontinuous transcription is 65 nucleotides wide. J. Virol..

[5] Kumar S., Nyodu R., Maurya V.K., Saxena S.K. (2020). Morphology, genome organization, replication, and pathogenesis of severe acute respiratory syndrome coronavirus 2 (SARS-CoV-2). Coronavirus Disease 2019 (COVID-19 23).

[6] Mariano G., Farthing R.J., Lale-Farjat S.L.M., Bergeron J.R.C. (2020). Structural characterization of SARS-CoV-2: Where we are, and where we need to be. Front. Mol. Biosci..

[7] Huang Y., Yang C., Xu X., Xu W., Liu S. (2020). Structural and functional properties of SARS-CoV-2 spike protein: Potential antivirus drug development for COVID-19. Acta Pharm. Sin..

[8] Zhu N., Zhang D., Wang W., Li X., Yang B., Song J., Zhao X., Huang B., Shi W., Lu R. (2020). A Novel Coronavirus from Patients with Pneumonia in China, 2019. N. Engl. J. Med..

[9] Gralinski L.E., Menachery V.D. (2020). Return of the Coronavirus: 2019-nCoV. Viruses.

[10] Beigel J.H., Tomashek K.M., Dodd L.E., Mehta A.K., Zingman B.S., Kalil A.C., Hohmann E., Chu H.Y., Luetkemeyer A., Kline S. (2020). Remdesivir for the treatment of Covid-19—final report. N. Engl. J. Med..

[11] Pan X., Dong L., Yang N., Chen D., Peng C. (2020). Potential drugs for the treatment of the novel coronavirus pneumonia (COVID-19) in China. Virus Res..

[12] Horby P., Lim W.S., Emberson J.R., Mafham M., Bell J.L., Linsell L., Staplin N., Brightling C., Ustianowski A., RECOVERY Collaborative Group (2021). Dexamethasone in Hospitalized Patients with Covid-19. N. Engl. J. Med..

[13] Nicola M., Alsafi Z., Sohrabi C., Kerwan A., Al-Jabir A., Iosifidis C., Agha M., Agha R. (2020). The socio-economic implications of the coronavirus pandemic (COVID-19): A review. Int. J. Surg..

[14] Zhao J., Zhao S., Ou J., Zhang J., Lan W., Guan W., Wu X., Yan Y., Zhao W., Wu J. (2020). COVID-19: Coronavirus Vaccine Development Updates. Front. Immunol..

[15] Burton D.R., Walker L.M. (2020). Rational Vaccine Design in the Time of COVID-19. Cell Host Microbe..

[16] Graham B.S. (2020). Rapid COVID-19 vaccine development. Science.

[17] Bhatta M., Nandi S., Dutta S., Saha M.K. (2021). Coronavirus (SARS-CoV-2): A systematic review for potential vaccines. Hum. Vaccin. Immunother..

[18] Huang H.Y., Wang S.H., Tang Y., Sheng W., Zuo C.J., Wu D.W., Fang H., Du Q., Li N. (2021). Landscape and progress of global COVID-19 vaccine development. Hum. Vaccin. Immunother..

[19] Kandeil A., Mostafa A., Hegazy R.R., El-Shesheny R., El Taweel A., Gomaa M.R., Shehata M., Elbaset M.A., Kayed A.E., Mahmoud S.H. (2021). Immunogenicity and Safety of an Inactivated SARS-CoV-2 Vaccine: Preclinical Studies. Vaccines.

[20] Wang H., Zhang Y., Huang B., Deng W., Quan Y., Wang W., Xu W., Zhao Y., Li N., Zhang J. (2020). Development of an Inactivated Vaccine Candidate, BBIBP-CorV, with Potent Protection against SARS-CoV-2. Cell.

[21] Gao Q., Bao L., Mao H., Wang L., Xu K., Yang M., Li Y., Zhu L., Wang N., Lv Z. (2020). Development of an inactivated vaccine candidate for SARS-CoV-2. Science.

[22] Tostanoski L.H., Wegmann F., Martinot A.J., Loos C., McMahan K., Mercado N.B., Yu J., Chan C.N., Bondoc S., Starke C.E. (2020). Ad26 vaccine protects against SARS-CoV-2 severe clinical disease in hamsters. Nat. Med..

[23] Munoz-Fontela C., Dowling W.E., Funnell S.G.P., Gsell P.-S., Riveros-Balta A.X., Albrecht R.A., Andersen H., Baric R.S., Carroll M.W., Cavaleri M. (2020). Animal models for COVID-19. Nature.

[24] Pandey K., Acharya A., Mohan M., Ng C.L., Reid S.P., Byrareddy S.N. (2021). Animal models for SARS CoV 2 research: A comprehensive literature review. Transbound. Emerg. Dis..

[25] Winkler E.S., Bailey A.L., Kafai N.M., Nair S., McCune B.T., Yu J., Fox J.M., Chen R.E., Earnest J.T., Keeler S.P. (2020). SARS-CoV-2 infection of human ACE2-transgenic mice causes severe lung inflammation and impaired function. Nat. Immunol..

[26] Yinda C.K., Port J.R., Bushmaker T., Offei Owusu I., Purushotham J.N., Avanzato V.A., Fischer R.J., Schulz J.E., Holbrook M.G., Hebner M.J. (2021). K18-hACE2 mice develop respiratory disease resembling severe COVID-19. PLoS Pathog..

[27] Pavel S.T.I., Yetiskin H., Aydin G., Holyavkin C., Uygut M.A., Dursun Z.B., Celik L., Cevik C., Ozdarendeli A. (2020). Isolation and characterization of severe acute respiratory syndrome coronavirus 2 in Turkey. PLoS ONE.

[28] Reed L.J., Muench H. (1938). A simple method of estimating fifty per cent endpoints. Am. J. Epidemiol..

[29] Mamedov T., Yuksel D., Ilgin M., Gurbuzaslan I., Gulec B., Yetiskin H., Uygut M.A., Pavel S.T.I., Ozdarendeli A., Mammadova G. (2021). Plant-Produced Glycosylated and In Vivo Deglycosylated Receptor Binding Domain Proteins of SARS-CoV-2 Induce Potent Neutralizing Responses in Mice. Viruses.

[30] Pavel S.T.I., Yetiskin H., Kalkan A., Ozdarendeli A. (2020). Evaluation of the cell culture based and the mouse brain derived inactivated vaccines against Crimean-Congo hemorrhagic fever virus in transiently immune-suppressed (IS) mouse model. PLoS Negl. Trop. Dis..

[31] Enkirch T., Von Messling V. (2015). Ferret models of viral pathogenesis. Virology.

[32] Marsh G.A., McAuley A.J., Au G.G., Riddell S., Layton D., Singanallur N.B., Layton R., Payne J., Durr P.A., Bender H. (2021). ChAdOx1 nCoV-19 (AZD1222) vaccine candidate significantly reduces SARS-CoV-2 shedding in ferrets. npj Vaccin..

[33] Kim Y.-I., Kim D., Yu K.-M., Seo H.D., Lee S.-A., Casel M.A.B., Jang S.-G., Kim S., Jung W., Lai C.-J. (2021). Development of Spike Receptor-Binding Domain Nanoparticles as a Vaccine Candidate against SARS-CoV-2 Infection in Ferrets. mBio.

[34] Wu F., Zhao S., Yu B., Chen Y.-M., Wang W., Song Z.-G., Hu Y., Tao Z.-W., Tian J.-H., Pei Y.-Y. (2020). A new coronavirus associated with human respiratory disease in China. Nature.

[35] Xiaoni C., Pengxiang W., Zhun W. (2021). Emergency use of COVID-19 vaccines recommended by the World Health Organization (WHO) as of June 2021. Drug Discov..

[36] Richman D.D. (2021). COVID-19 vaccines: Implementation, limitations and opportunities. Glob. Health Med..

[37] Massinga Loembé M., Nkengasong J.N. (2021). COVID-19 vaccine access in Africa: Global distribution, vaccine platforms, and challenges ahead. Immunity.

[38] Tai W., He L., Zhang X., Pu J., Voronin D., Jiang S., Zhou Y., Du L. (2020). Characterization of the receptor-binding domain (RBD) of 2019 novel coronavirus: Implication for development of RBD protein as a viral attachment inhibitor and vaccine. Cell. Mol. Immunol..

[39] Forni G., Mantovani A. (2021). COVID-19 vaccines: Where we stand and challenges ahead. Cell Death Differ..

[40] Chen R.E., Zhang X., Case J.B., Winkler E.S., Liu Y., VanBlargan L.A., Liu J., Errico J.M., Xie X., Suryadevara N. (2021). Resistance of SARS-CoV-2 variants to neutralization by monoclonal and serum-derived polyclonal antibodies. Nat. Med..

[41] Wang G.-L., Wang Z.-Y., Duan L.-J., Meng Q.-C., Jiang M.-D., Cao J., Yao L., Zhu K.-L., Cao W.-C., Ma M.-J. (2021). Susceptibility of Circulating SARS-CoV-2 Variants to Neutralization. N. Engl. J. Med..

[42] Wang P., Nair M.S., Liu L., Iketani S., Luo Y., Guo Y., Wang M., Yu J., Zhang B., Kwong P.D. (2021). Antibody resistance of SARS-CoV-2 variants B.1.351 and B.1.1.7. Nature.

[43] Kulkarni R. (2019). Antibody-Dependent Enhancement of Viral Infections. Dynamics of Immune Activation in Viral Diseases.

[44] Deming D., Sheahan T., Heise M., Yount B., Davis N., Sims A., Suthar M., Harkema J., Whitmore A., Pickles R. (2006). Vaccine efficacy in senescent mice challenged with recombinant SARS-CoV bearing epidemic challenged with recombinant SARSCoV bearing epidemicand zoonotic spike variants. PLos. Med..

[45] Yasui F., Kai C., Kitabatake M., Inoue S., Yoneda M., Yokochi S., Kase R., Sekiguchi S., Morita K., Hishima T. (2008). Prior immunization with severe acut erespiratory syndrome (SARS)-associated coronavirus (SARS-CoV) nucleocapsid protein causes severe pneumoniain mice infected with SARS-CoV. J. Immunol..

[46] Wang S.F., Tseng S.P., Yen C.H., Yang J.Y., Tsao C.H., Shen C.W., Chen K.H., Liu F.T., Liu W.T., Chen Y.M. (2014). Antibody-dependent SARS coronavirus infection is mediated by antibodies against spike proteins. Biochem. Biophys. Res. Commun..

[47] Luo F., Liao F.L., Wang H., Tang H.B., Yang Z.Q., Hou W. (2018). Evaluation of antibody- dependent enhancement of SARS- CoV infection in rhesus macaques immunized with an inactivated SARS- CoV vaccine. Virol. Sin..

[48] Liu L., Wei Q., Lin Q., Fang J., Wang H., Kwok H., Tang H., Nishiura K., Peng J., Tan Z. (2019). Anti-spike IgG causes severe acute lung injury by skewing macrophage responses during acute SARS-CoV infection. Jci. Insight.

[49] Kaabi N.A., Zhang Y., Xia S., Yang Y., Qahtani M.M.A., Abdulrazzag N., Nusair M.A., Hassani M., Jawad J.S., Abdalla J. (2021). Effect of 2 Inactivated SARS-CoV-2 Vaccines on Symptomatic COVID-19 Infection in Adults A Randomized Clinical Trial. JAMA.

[50] Krammer F. (2020). SARS-CoV-2 vaccines in development. Nature.

[51] Poland G.A., Ovsyannikova I.G., Kennedy R.B. (2020). SARS-CoV-2 immunity: Review and applications to phase 3 vaccine candidates. Lancet.

[52] Barrett P.N., Terpening S.J., Snow D., Cobb R.R., Kistner O. (2017). Vero cell technology for rapid development of inactivated whole virus vaccines for emerging viral diseases. Exp. Rev. Vaccin..

[53] Sanders B., Koldijk M., Schuitemaker H. (2015). Inactivated Viral Vaccines’. Vaccine Analysis: Strategies, Principles, and Control.

[54] Ragan I.K., Hartson L.M., Dutt T.S., Obregon-Henao A., Maison R.M., Gordy P., Fox A., Karger B.R., Cross S.T., Kapuscinski M.L. (2021). A Whole Virion Vaccine for COVID-19 Produced via a Novel Inactivation Method and Preliminary Demonstration of Efficacy in an Animal Challenge Model. Vaccines.

[55] Marrack P., McKee A.S., Munks M.W. (2009). Towards an understanding of the adjuvant action of aluminium. Nat. Rev. Immunol..

[56] He P., Zou Y., Hu Z. (2015). Advances in aluminum hydroxide-based adjuvant research and its mechanism. Hum. Vaccin. Immunother..

[57] Ghimire T.R. (2015). The mechanisms of action of vaccines containing aluminum adjuvants: An in vitro vs. in vivo paradigm. Springerplus.

[58] Hotez P.J., Corry D.B., Strych U., Bottazzi M.E. (2020). COVID-19 vaccines: Neutralizing antibodies and the alum advantage. Nat. Rev. Immunol..

[59] Robbiani D.F., Gaebler C., Muecksch F., Lorenzi J.C.C., Wang Z., Cho A., Agudelo M., Barnes C.O., Gazumyan A., Finkin S. (2020). Convergent antibody responses to SARS-CoV-2 in convalescent individuals. Nature.

[60] Brouwer P.J.M., Caniels T.G., van der Straten K., Snitselaar J.L., Aldon Y., Bangaru S., Torres J.L., Okba N.M.A., Claireaux M., Kerster G. (2020). Potent neutralizing antibodies from COVID-19 patients define multiple targets of vulnerability. Science.

[61] Baldwin W.R., Livengood J.A., Giebler H.A., Stovall J.L., Boroughs K.L., Sonnberg S., Bohning K.J., Dietrich E.A., Ong Y.T., Danh H.K. (2018). Purified Inactivated Zika Vaccine Candidates Afford Protection against Lethal Challenge in Mice. Sci. Rep..

[62] Yao Y.-F., Wang Z.-J., Jiang R.-D., Hu X., Zhang H.-J., Zhou Y.-W., Gao G., Chen Y., Peng Y., Liu M.-Q. (2021). Protective Efficacy of Inactivated Vaccine against SARS-CoV-2 Infection in Mice and Non-Human Primates. Virol. Sin..

[63] Ganneru B., Jogdand H., Daram V.K., Das D., Molugu N.R., Prasad S.D., Kannappa S.V., Ella K.M., Ravikrishnan R., Awasthi A. (2021). Th1 skewed immune response of whole virion inactivated SARS CoV 2 vaccine and its safety evaluation. Iscience.

[64] Gerdts V., Wilson H.L., Meurens F., van Drunen Littel-van den Hurk S., Wilson D., Walker S., Wheler C., Townsend H., Potter A.A. (2015). Large animal models for vaccine development and testing. Ilar J..

[65] Mukhopadhyay L., Yadav P.D., Gupta N., Mohandas S., Patil D.Y., Shete-Aich A., Panda S., Bhargava B. (2021). Comparison of the immunogenicity & protective efficacy of various SARS-CoV-2 vaccine candidates in non-human primates. Ind. J. Med. Res..

[66] Monchatre-Leroy E., Lesellier S., Wasniewski M., Picard-Meyer E., Richomme C., Boue F., Lacote S., Murri S., Pulido C., Vulin J. (2021). Hamster and ferret experimental infection with intranasal low dose of a single strain of SARS-CoV-2. J. Gen. Virol..

[67] Kim Y.I., Kim S.G., Kim S.M., Kim E.H., Park S.J., Yu K.M., Chang J.H., Kim E.J., Lee S., Casel M.A.B. (2020). Infection and Rapid Transmission of SARS-CoV-2 in Ferrets. Cell Host Microbe.

[68] Everett H.E., Lean F.Z.X., Byrne A.M.P., van Diemen P.M., Rhodes S., James J., Mollett B., Coward V.J., Skinner P., Warren C.J. (2021). Intranasal Infection of Ferrets with SARS-CoV-2 as a Model for Asymptomatic Human Infection. Viruses.

[69] Ryan K.A., Bewley K.R., Fotheringham S.A., Slack G.S., Brown P., Hall Y., Wand N.I., Marriott A.C., Cavell B.E., Tree J.A. (2021). Dose-dependent response to infection with SARS-CoV-2 in the ferret model and evidence of protective immunity. Nat. Commun..

[70] Pollard A.J., Bijker E.M. (2021). A guide to vaccinology: From basic principles to new developments. Nat. Rev. Immunol..

[71] Zhang Y., Zeng G., Pan H., Li C., Hu Y., Chu K., Han W., Chen Z., Tang R., Yin W. (2020). Safety, tolerability, and immunogenicity of an inactivated SARS-CoV-2 vaccine in healthy adults aged 18–59 years: A randomised, double-blind, placebo-controlled, phase 1/2 clinical trial. Lancet Infect. Dis..

[72] Deng Y., Li Y., Yang R., Tan W. (2021). SARS-CoV-2-specific T cell immunity to structural proteins in inactivated COVID-19 vaccine recipients. Cell Mol. Immunol..

[73] Hotez P.J., Corry D.B., Bottazzi M.E. (2020). COVID-19 vaccine design: The Janus face of immune enhancement. Nat. Rev. Immunol..

