# Supplementary material for: Development of an Inactivated Vaccine against SARS CoV-2
Source: Vaccines (Basel). 2021 Nov 2;9(11):1266. doi: 10.3390/vaccines9111266 (PMC8624180; doi:10.3390/vaccines9111266)
Supplement: Supplementary file 1 [file vaccines-09-01266-s001.zip › vaccines-1429720-supplementary.pdf]

vaccines

MDPI

Supplementary

A B

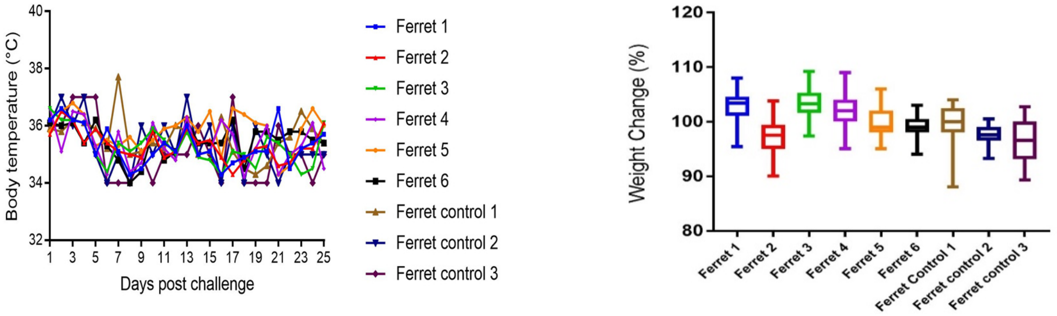

Figure S1. Body temperature and body weight were recorded in ferrets every 2 days when administered N+1 (6 ugr Ag and Alhydrogel) dose regimen. Control animals received N+1 saline buffer. There were no obvious body temperatures and body weight changes during this period in all animals.

Negative control group 6 ugr dose group

A

B

C

D

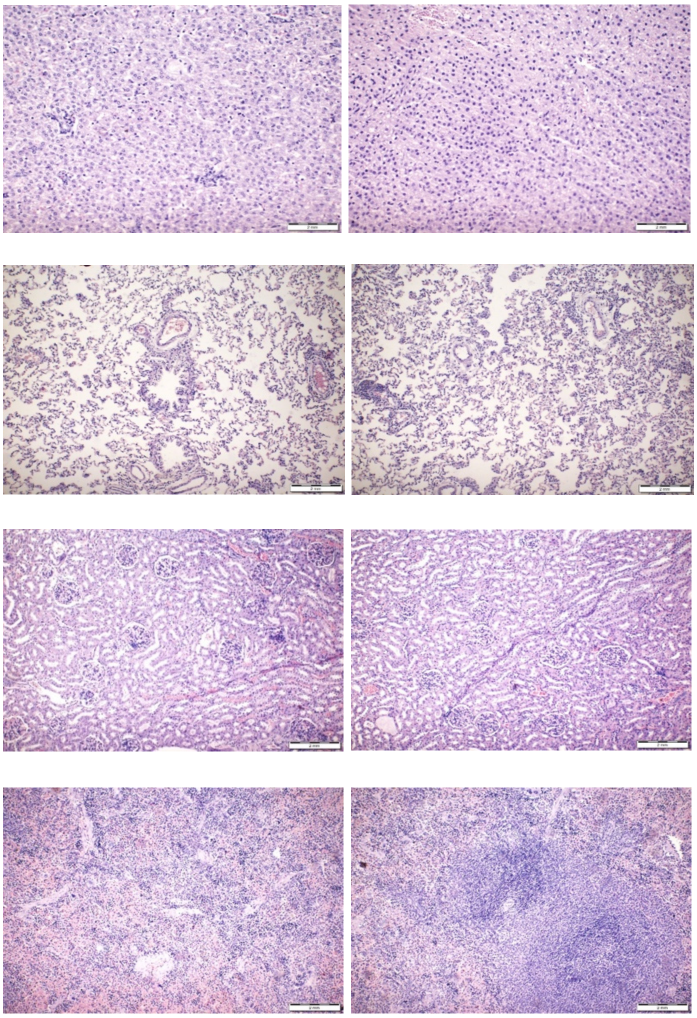

Figure S2. Representative Hematoxylin and Eosin-stained liver (A), lung (B), kidney (C) and spleen (D) tissues from ferrets, when administered with saline buffer and adjuvanted vaccine (6 ugr Ag and Alhydrogel). There were no pathological changes in these main organs. Scale bar is 2 mm, HE ×100.

Table S1: List of detected variants in hCoV-19/Turkey/ERAGEM-001/2020 strain in Passage 3 (P3) and Passage 10 (P10) compared to the MN908947.3 reference genome of a SARS-CoV-2 Wuhan isolate. Comparison of hCoV-19/Turkey/ERAGEM-001/2020 strain and the MN908947.3 reference genome of a SARS-CoV-2 Wuhan isolate was published from our group (see ref. [24]).

Pos	Gene	Ref	P3	P10	AAPos	RefAA	P3AA	P10AA	Depth	VF(%) *
1397	ORF1ab	G	A	A	378	Val	Ile	Ile	45501	99.6
11083	ORF1ab	G	T	T	3606	Leu	Leu	Leu	17037	72.3
22213	S	T	T	C	217	Pro	Pro	Pro	9952	64.7
23876	S	G	A	A	772	Val	Ile	Ile	21239	99.9
28688	N	T	C	C	139	Leu	Leu	Leu	35885	99.7
29563	ORF10	C	T	T	2	Gly	Gly	Gly	55600	99.4
29742	3′UTR	G	T	T	-	-	-	-	78352	99.7

* VF(%): Variant fraction
